# New Insights on the Role of pentraxin-3 in Allergic Asthma

**DOI:** 10.3389/falgy.2021.678023

**Published:** 2021-06-11

**Authors:** Latifa Koussih, Samira Atoui, Omar Tliba, Abdelilah S. Gounni

**Affiliations:** ^1^Department of Immunology, Max Rady College of Medicine, Rady Faculty of Health Sciences, University of Manitoba, Winnipeg, MB, Canada; ^2^Department des Sciences Experimentales, Universite de Saint-Boniface, Winnipeg, MB, Canada; ^3^Department of Biomedical Sciences, College of Veterinary Medicine, Long Island University, Brookville, NY, United States

**Keywords:** pentraxin-3, allergic asthma, innate immunity, airway remodeling, glucocorticosteroids

## Abstract

Pentraxins are soluble pattern recognition receptors that play a major role in regulating innate immune responses. Through their interaction with complement components, Fcγ receptors, and different microbial moieties, Pentraxins cause an amplification of the inflammatory response. Pentraxin-3 is of particular interest since it was identified as a biomarker for several immune-pathological diseases. In allergic asthma, pentraxin-3 is produced by immune and structural cells and is up-regulated by pro-asthmatic cytokines such as TNFα and IL-1β. Strikingly, some recent experimental evidence demonstrated a protective role of pentraxin-3 in chronic airway inflammatory diseases such as allergic asthma. Indeed, reduced pentraxin-3 levels have been associated with neutrophilic inflammation, Th17 immune response, insensitivity to standard therapeutics and a severe form of the disease. In this review, we will summarize the current knowledge of the role of pentraxin-3 in innate immune response and discuss the protective role of pentraxin-3 in allergic asthma.

## Introduction

Asthma is a chronic airway inflammatory disease characterized by episodes of acute bronchoconstriction and manifests various typical symptoms such as coughing, shortness of breath, wheezing, and chest tightness (1). Although it is still unclear what causes asthma, it is well-known that asthma attacks can be triggered by allergens, tobacco smoke, occupational hazards, or even exercise. Interestingly, an increase in atopic sensitization along with allergic conditions such as rhinitis and eczema were reported to correlate with asthma prevalence (2).

Susceptibility to asthma is likely due, at least in part, to genetic factors. There are four major groups of asthma susceptibility genes: (i) genes associated with innate immunity and immuno-regulation: this category refers to genes encoding pattern recognition receptors (PRRs), which are surface receptors involved in the binding of molecules frequently found in pathogens or released by damaged cells; (ii) genes associated with Th2-cell differentiation and effector functions including genes encoding pro-inflammatory cytokines involved in the maturation of specific T cell responses (3); (iii) genes associated with epithelial cell functions as well as mucosal immunity (4–8) and (iv) genes associated with lung function, airway remodeling and disease severity (3) such as genes encoding adrenergic beta receptor 2 (ADRB2) (9), extracellular matrix protein tenascin c (10), as well as pro-inflammatory molecules such as tumor necrosis factor (TNF) (11), leukotriene C4 synthase (12), and TGF-β1 (13). These genes are highly heterogeneous and have a marked impact on lung physiology and phenotype. Besides genetic factors, epigenetic factors impact the development and progression of the lung's pathology. They may appear even before asthma symptoms. Examples of these modifications include methylation of the promoter and intronic regions of the IL-4 gene (14) and hyper-methylation of the IFN-γ gene (15).

Despite the central role that genetic and epigenetic factors play in determining the outcome of the disease, they act hand in hand with environmental factors adding thereby another layer of complexity in the pathogenesis of asthma. These factors include respiratory viruses, tobacco smoke, air pollutants, toxins, and allergens, which exacerbate allergic asthma (16). However, other studies highlighted the importance of environmental factors in protecting against the development of asthma. Indeed, the “Hygiene Hypothesis” concept states that exposure to allergens or an environment rich in microbes at an early age may be beneficial and may contribute to the development of immunity against allergic diseases later in life (1, 17, 18).

Mechanistically, previous studies have highlighted the role of adaptive immunity in regulating allergic asthma responses and in guiding several therapeutic approaches to overcome asthma symptoms. However, in the past two decades, attention has been shifted toward the critical role that the innate, non–antigen-dependent immune system plays in asthma pathogenesis. This review will highlight the general structure and expression of pentraxins-3, its crucial role in innate immunity, and the potential links to allergic asthma.

## Innate Immune Response: Role of PRRs

Innate immunity is the first line of defense consisting of white blood cells, skin and mucous membranes, and body fluids. Innate immune responses are rapid but non-specific and transient (19). The innate and adaptive immunity work together and are tightly integrated as a single defense (19). The adaptive immune response is initiated, activated, and regulated by the innate immune response through antigen presentation and cytokine production (20). Although innate immunity is further divided into efferent and afferent arms, the afferent arm is critical due to its ability to identify specific molecular patterns on pathogens (21).

Pattern recognition receptors (PRR) are critical elements of the innate immune system and play an essential role in allergic inflammation. PRRs are expressed by epithelial cells, dendritic cells, macrophages, and other immune and non-immune cells. They recognize PAMPs expressed by microorganisms and Damage Associated Molecular Patterns (DAMPs) that consist of cell components released during cell damage or death (22–24). PAMPs recognized by PRRs include carbohydrates, nucleic acids, peptides peptidoglycans, *N*-formylmethionine, lipoproteins and fungal glucans and chitin, while DAMPS include uric acid and extracellular ATP.

The PRRs of the innate immune system are divided into four families: the membrane-bound Toll-like receptors (TLRs), C-type lectin receptors (CLRs), retinoic acid-inducible gene-1-like receptors (RLRs), and NOD-like receptors (NLRs; nucleotide-binding oligomerization domain receptors) (25). These receptors are expressed at the cell surface and recognize extracellular pathogens. However, some PPRs could be found inside the cell, either in the cytoplasm or in endosomes. They can recognize/sense intracellular invaders, such as viruses, or in a soluble form circulating in the body fluids.

The cell-bound PRRs are further classified into endocytic PRRs and signaling PRRs. Endocytic PRRs are involved in microorganism attachment, engulfment, uptake, and destruction and induce phagocytosis and inflammation. Signaling PRRs relay intracellular signals upon contact with pathogens and activate various killing mechanisms (26). Soluble PRRs, such as collectins, ficolins, complement components, and pentraxins, which constitute the humoral/afferent arm of the innate immunity, are of particular importance in asthma.

## Pentraxins Superfamily

Pentraxins, so named because they are assembled in pentamers, are soluble PRRs that play an essential role in immunological responses. This family of multifunctional proteins is classified into long and short pentraxins; both evolutionary conserved in human, mouse, rat, opossum, chicken and some lower vertebrates (27). Short pentraxins consist of C-reactive protein (CRP) and serum amyloid P (SAP) and are produced mainly by hepatocytes (28, 29). Long pentraxins are larger proteins and include pentraxin-3, pentraxin-4, neuronal pentraxin-1 (NP1) and NP2. Pentraxin-3 is of particular interest since it was identified as a biomarker for several pathological states and has been proposed to play a protective role in various diseases (30).

## Pentraxin-3 Gene Structure

The human and murine pentraxin-3 genes are localized on a syntenic region of chromosome 3 band q25 (31, 32). Pentraxin-3 gene is organized into promoter region and three exons: the first exon encodes for the signal peptide, the second exon encodes for the N-terminal domain, whereas the third exon encodes for the C-terminal domain matching exactly the second exon of the short pentraxin genes (32, 33). The mouse homolog, murine pentraxin-3, displays high homology with human pentraxin-3 both at the gene (92%) and protein levels (82%) (29, 34). Such homology makes the mouse an excellent model to study the functions of human pentraxin-3 *in vivo* (35).

The sequence of the 5′-flanking region of the *human* pentraxin*-3* gene reveals features of a eukaryotic promoter. It contains several potential binding sites for transcription factors, including NF-IL 6, NF-κB, activator protein 1 (AP-1) and AP-2, Pu.1, polyomavirus enhancer A (PEA-3), E 26 transformation specific sequence-1 (Ets-1), specificity protein (Sp1), glucocorticoid responsive elements (GRE) and gamma interferon activation site (GAS) binding sites (36, 37). The *murine* pentraxin-*3* promoter displays only 50.1% homology with *human* pentraxin-*3* counterpart (37). In addition to DNA binding sites present in the *human* pentraxin*-3* promoter, *murine* pentraxin*-3* gene's promoter contains binding sites for Hox-1.3 (38).

Additional differences were also reported between short and long pentraxins. Unlike short pentraxins, the pentraxin-3 promoter does not contain a consensus binding site for hepatic nuclear factor-1 (HNF-1), which may explain why long pentraxins such as pentraxin-3 are not induced in the liver (38). Altogether, these studies indicate the species differences in pentraxin-3 gene and promoter architectures and further studies are needed to understand the functional consequences of such differences.

## Pentraxin-3 Protein Structure

Pentraxin superfamily is an evolutionarily conserved family of proteins. It is characterized by a pentameric structure made up of five monomers with radial symmetry (29). Pentraxin superfamily is divided into two subclasses based on their size. The classical short pentraxins that consist of C-reactive protein (CRP) in humans and serum amyloid P component (SAP) in mice are acute-phase proteins produced in the liver in response to inflammatory mediators, particularly IL-6 (39, 40). They have a quaternary structure made of five identical subunits held together by non-covalent interactions. The long pentraxins are characterized by an evolved N-terminal domain coupled to a pentraxin-like C-terminal domain (41). The prototypic long pentraxins is pentraxin-3, a homo-octameric secreted glycoprotein (34). The human pentraxin-3 protein is composed of 42 kDa subunits (32, 42) and arranged as octamers made from two tetramers held together by covalent bonds (29, 43, 44). In addition to the conserved C-terminal domain common to all pentraxins, pentraxin-3 contains a unique N-terminal region-specific to long pentraxins (45). The N terminal domain secondary structure consists of four helices (αA, αB, αC, and αD) connected with short loops, three of which, αB, αC, and αD helices, form coiled-coil conformation (32, 44, 46). While no experimental 3D model of pentraxin-3 has been published up to now, homology models proposed a secondary structure of pentraxin-3 (32, 46, 47). In such models, the C-terminal domain presents a hydrophobic core and is formed by two antiparallel β sheets, stabilized by Cys 210 and 271 organized into a β-jelly roll topology (39, 48) and a single α helix located on the protein surface (46, 49). In addition to its multimeric organization, the human pentraxin-3 protein forms a unique asymmetric complex with an oligomeric quaternary structure composed of eight identical protomer subunits that form octamers (44, 50).

Mature pentraxin-3 molecular weight is predicted to be 42 kDa however; N-glycosylation at Asn220 in the C-terminal domain increases the molecular weight to 45 kDa (29). This single N-glycosylation site of pentraxin-3 is occupied by complex-type oligosaccharides, mainly fucosylated and sialylated biantennary moieties (51). Pentraxin-3 glycosylation is essential in inflammation, albeit it can be affected in a cell and stimulus-specific manner. Besides, differences in the level of pentraxin-3 glycosylation is associated with the pathogenesis of various diseases (52). This effect is mainly mediated through pentraxin-3 glycosylated motifs interaction with C1q, which is necessary to activate the classical and alternative complement pathway (52).

Proteolytic cleavage of pentraxin-3 domains dramatically affects its regulation and expression. While the N-terminal domain of pentraxin-3 is critical in protein multimerization, a recent study conducted by Delneste et al. showed that such domain is susceptible to cleavage by proteases within minutes, particularly by neutrophil elastase and *Aspergillus fumigatus* proteases (53). The rapid cleavage of N-terminal domain may be due to the presence of *A. fumigatus* binding site in such domain (54). However, due to the lack of specific inhibitors, it is still unclear which *A. fumigatus* serine protease(s) are involved in the degradation of pentraxin-3 (35, 53).

## Pentraxin-3 Cellular Sources And Modulation By Inflammatory Mediators And Glucocorticoids

Pentraxin-3 can be produced by immune and non-immune cells (33, 55) (Table 1). Among immune cells, myeloid cells, especially DCs, are the primary sources of pentraxin-3 (34), where the expression of pentraxin-3 is differentially modulated by inflammatory mediators such as cytokines, TLR ligands, and CD40L. For instance, IL-10 has been shown to enhance the ability of LPS to induce pentraxin-3 expression. However, IFN-γ, IL-4, prostaglandin E2, dexamethasone, and vitamin D3 inhibit pentraxin-3 expression in DCs (56, 57). Unlike myeloid DCs, plasmacytoid DCs have not been shown to express pentraxin-3. Similar to DCs, mononuclear macrophages/phagocytes express pentraxin-3 when exposed to microbial pathogens (58). Monocytes also produce pentraxin-3 upon stimulation with IL-1β, TNF, LPS, and lipoarabinomannans (32, 49).

**Table 1:** Cellular source of pentraxin-3 in the lung.

	**Inducing factors**	**Suppressing factors**
Airway epithelial cells	IL-1β, TNF	
Fibroblasts	IL-1β, TNF, LPS, GCs	
Airway smooth muscle cells	IL-1β, TNF, GCs	
Endothelial cells	IL-1β, TNF, GCs, LPS, oxidized LDL	
Neutrophils	IL-1β, TNF, thrombin, TLR agonists, microbes, and DAMPs /PAMPS	
Macrophages/Monocytes	IL-1β, TNF, thrombin, TLR agonists, microbes, and DAMPs /PAMPS	IFNγ
Myeloid dendritic cells	IL-1β, IL-10, TLR agonists, microbes, and DAMPs /PAMPS	IFNγ, GCs, IL-4, PGE2, Vitamin-D3

Neutrophils are the only granulocytes that act as pentraxin-3 reservoirs. Unlike the *de-novo* synthesis of pentraxin-3 in mononuclear phagocytes and DCs, pentraxin-3 in neutrophils are stored in granules in a soluble form (54). After exposure to microbial pathogens and in response to TLR agonists, neutrophils rapidly release the monomeric pentraxin-3 from their myeloperoxidase (MPO) negative granules. While parts of the released pentraxin-3 interact with each other and form multimers, others remain attached to neutrophils extracellular trap (NET) and generate an effective anti-microbial microenvironment (41, 57). Unlike the innate immune cells, the adaptive immune cells such as lymphocytes, T and B cells, and natural killer cells do not express pentraxin-3, further highlighting the importance of pentraxin-3 in innate immunity (58).

Besides innate immune cells, structural cells also express pentraxin-3. In lung airway epithelial cells (59, 60), and human proximal renal tubular epithelial cells, pentraxin-3 expression is induced by mechanical stretch, LPS, CD40L, and cytokines such as IL-1β, TNF, and IL-17 (61). In synoviocytes from patients with rheumatoid arthritis, pentraxin-3 expression was synergistically induced by IL-1β and Oncostatin M (OSM) (62), not affected by TNF or IL-1β, and dramatically reduced by TGF-β and IFN-γ (49). In vascular endothelium and smooth muscle cells, pro-inflammatory signals including IL-1β, LPS, oxidized LDL, and the products of their degradation stimulate pentraxin-3 synthesis and production at the site of infection. Pentraxin-3 synthesis at the vascular wall of endothelial cells is up-regulated by HDL, allowing pentraxin-3 to act in an autocrine/paracrine manner to regulate vascular endothelium function (32, 63, 64). While pentraxin-3 is generally not detected in brain cells, inflammatory stimuli such as LPS, TNF, and IL-1β induce pentraxin-3 synthesis in granule cells, presumptive glial cells in the white matter (corpus callosum, fimbria), meningeal pia mater, and dentate gyrus hilus (65, 66). Altogether, these studies suggest that pentraxin-3 expression and synthesis is regulated in a cell and stimulus-specific manner.

Several studies examined the effects of anti-inflammatories such as glucocorticoids (GCs) on pentraxin-3 expression. *In vivo* studies, for instance, showed divergent effects of GCs on pentraxin-3 expression. When dexamethasone was administered prophylactically to children undergoing open-heart surgery, Lerzo et al. (67) showed a significant increase in the blood pentraxin-3 levels. Such increase of pentraxin-3 mediated several beneficial effects of GCs in these patients, such as decreasing inflammatory parameters or improving clinical outcomes. Similarly, Doni et al. found that *in vivo* administration of GCs augmented the blood levels of pentraxin-3 in mice (68). Interestingly, patients with Cushing syndrome had an increased circulating level of pentraxin-3, whereas pentraxin-3 levels were decreased in subjects affected by iatrogenic hypocortisolism (68). Conversely, Pulsatelli et al. (69) failed to show any effect of corticosteroid therapy on circulating pentraxin-3 levels in patients with polymyalgia rheumatica. Together, the *in vivo* effect of GCs on pentraxin-3 expression is largely disease-specific, where GCs tend to enhance the circulating levels of pentraxin-3 in most cases.

Interestingly, several *in vitro* studies also showed a divergent effect of GCs on pentraxin-3 expression. For example, while proteomic analysis of peripheral blood mononuclear cells (PBMCs) showed no effect of dexamethasone on pentraxin-3 expression (70), GCs enhance the ability of LPS to induce pentraxin-3 expression in macrophages (71) by promoting the recruitment of GC receptor (GR) to the NF-κB binding sites in pentraxin-3 promoter. Interestingly, additional studies in structural cells showed positive effects of GCs in pentraxin-3 expression. For example, when fibroblasts derived from patients with major depression were treated with dexamethasone, a significant increase of pentraxin-3 was seen (72). In line with this, we recently showed that dexamethasone increases the expression of pentraxin-3 in human ASM cells in a p42/p44 ERK-dependent manner (73). However, other studies showed opposing effects of GCs on pentraxin-3 expression in immune vs. non-immune/structural cells. For instance, while in myeloid cells, GCs have been shown to inhibit the expression of pentraxin-3. In fibroblasts and endothelial cells, GCs enhance pentraxin-3 production (68). The authors further demonstrated that GR repressed pentraxin-3 gene transcription by interfering with the action of other signaling pathways such as NF-κB and AP-1 in myeloid cells, while in fibroblasts and endothelial cells, GR functioned as a ligand-dependent transcription factor to induce pentraxin-3 gene expression. Together, GC's divergent effects on pentraxin-3 expression/production probably reflect the different functions of this multifunctional molecule in innate immunity.

## Pentraxin-3 Ligands

Pentraxin-3 is a specific pattern recognition receptor capable of binding to a variety of ligands. In some cases, ligand binding is mediated by the N-terminal domain tetramers. In such a case, the N-terminal domain acts as functional units critical in recognition of specific ligands involved in cumulus oophorus extracellular matrix organization and angiogenesis/anti-restenosis such as inter-α-inhibitor (IαI) and Fibroblast Growth Factor 2 (FGF2), respectively (74, 75). For example, the binding to FGF-2 to pentraxin-3 N-terminal domain (74) allows pentraxin-3 to inhibit FGF-2 induced-proliferation in vascular smooth muscle cells and endothelial cells (76, 77).

Additional pentraxin-3 ligands include (i) the complement fraction C1q and Factor H (FH), critical in the activation of the classical and alternative complement pathway, respectively (78); (ii) ficolin- 1 (M-ficolin) and mannose-binding lectin (MBL) involved in the activation of the lectin complement pathway (79, 80); (iii) P-selectin involved in the regulation of inflammatory processes and leukocyte recruitment (81); (iv) TNF-induced protein 6 (TSG6) involved in extracellular matrix organization and remodeling during inflammation and has a positive effect on female fertility (46, 54, 82). Since TSG6 binds pentraxin-3 at its N-terminal domain, the same binding site as FGF-2, it competes with FGF-2 and interferes with the inhibitory effect of pentraxin-3 on FGF-2 mediated angiogenesis (82); and vi) Bacterial OmpA of *Klebsiella pneumoniae* (KpOmpA), an inflammatory mediator involved in microbial recognition and innate immunity (43, 46). Together, several ligands can bind to pentraxin-3, further diversifying its biological effects in health and disease.

## Pentraxin-3: Mechanism of Action

The first mechanism of action is based on the interaction of pentraxin-3 with complement components. Interestingly, the complement fraction C1q was the first described pentraxin-3 ligand (43, 61). C1q is a component of the C1 complex involved in the classical complement pathway, a system of plasma proteins that enables antibodies and phagocytic cells to remove pathogens and damaged cells in three sequential steps that consist of phagocytosis, inflammation, and plasma membrane attack (83). Through its C-terminal domain, pentraxin-3 binds to the pentraxin-3-binding domain of C1q located in its globular head region (61, 84) and triggers the activation of the downstream cascade. However, such interaction could be weakened by other pentraxins, which may compete with pentraxin-3 to bind C1q (42). Besides, pentraxin-3 glycosylation may also reduce such interaction (52), while removal of sialic acid or complete deglycosylation of pentraxin-3 significantly enhances its binding to C1q (52). Moreover, whether pentraxin-3 is free or attached to the extracellular matrix differentially modulates its ability to activate the complement system (42). Free circulating pentraxin-3 inhibits C1q's interaction with immunoglobulins and prevents the activation of other complement system components and the formation of C1q/immunoglobulin complexes (85). However, extracellular matrix-attached pentraxin-3 binds to the surface of apoptotic cells, along with complement fractions C3b/iC3b bound to the pathogen surface, and promotes opsonization and the activation of the effector cascade (83).

Pentraxins can also interact with other components of the complement systems. For instance, Jarva et al. showed that the short pentraxin, CRP interacts with the regulatory protein factor H (FH) (35, 58), a key regulator of the alternative pathway of the complement (86, 87). Interestingly, CRP has been shown to bind components of the hyphal wall of *Aspergillus fumigatus*, and promotes phagocytosis, yet whether the alternative pathway of the complement is involved still needs further investigation (88). In addition to FH, Moalli et al. showed a critical role of another key regulator of the alternative pathway of the complement, Factor B, in mediating pentraxin-3 activities (54). The authors found that pentraxins-3-dependent phagocytosis of conidia requires Factor B (54). While the pro-phagocytic activity of endogenous pentraxin-3 was maintained in the absence of C1q, C4, and C1q, such action was impaired in the absence of the Factor B. Strikingly, the *in vitro* reconstitution of the alternative pathway (e.g., Factor B) was sufficient to restore pentraxin-3 activity (54).

Further, pentraxin-3 can also interact with mannan-binding lectin (MBL), which binds carbohydrate moieties on pathogen surfaces and activates the complement's lectin pathway. MBL-pentraxin-3 heterocomplexes can then recruit C1q, allowing communication between the classical and lectin complement pathways (56). Similarly, pentraxin-3 can bind another component of the lectin pathway: ficolins (79). The terminal sialic acid moieties of the pentraxin-3 N-domain bind M-ficolin through their fibrinogen-binding domain, forming M-ficolin–pentraxin-3 complexes (79). Ficolins recognize the sugar residues on the surface of the pathogen or dying cells, activate the lectin pathway, and prime the adaptive immune response (89).

The second mechanism of action is based on the interaction of pentraxin-3 with Fcγ receptors. FcγRI (CD64) and FcγRII (CD32) are two IgG receptors that have been shown to mediate the interaction between the short pentraxins such as CRP and SAP and the phagocytic cells. While FcγRII, the low-affinity IgG receptor, has a high affinity to CRP and provides stimulatory signals to cells, FcγRI, the high-affinity IgG receptor, has a low-affinity to CRP and provides inhibitory signals to cells. While IgG and CRP may compete for the same binding site on the FcγR, it is still unclear whether such competition affects immune responses (90). Interestingly, pentraxins-3 has been shown to recognize FcγRs and specifically FcγRIII (90). Additional studies showed that pentraxin-3, acting as an opsonin, facilitates conidia recognition and phagocytosis in an FcγR-dependent manner (54). The authors also found that the protective activity of exogenous pentraxin-3 against *Aspergillus fumigatus* is abrogated in FcγR-deficient mice. These findings demonstrated the functional involvement of FcγRs in pentraxin-3-dependent opsono-phagocytosis of Aspergillus fumigatus conidia by phagocytic cells (54). However, pentraxin-3 may interact with additional Fc receptors, which explains its diverse cellular functions.

The third mechanism of action is based on pentraxin-3's ability to recognize and interact with different microbial moieties, causing an amplification of the inflammatory response. For example, pentraxin-3 binds, with high affinity, to bacterial components of the outer membrane protein A of the Gram-negative bacteria, *K. pneumoniae* (KpOmpA) (91). Further evidence demonstrated that pentraxin-3 amplifies the inflammatory response to KpOmpA through complement activation (91). Besides, pentraxin-3 has been shown to bind to other bacteria, whether Gram-positive or Gram-negative, such as *Staphyloccocus aureus, Pseudomonas aeruginosa, Salmonella typhimurium, Streptococcus pneumoniae*, and *Neisseria meningitidis* and viruses such as cytomegalovirus (CMV) and H3N2 influenza virus (88, 92, 93). Pentraxin-3's binding to these microbial moieties facilitates cytokine production at inflammatory sites and increases the uptake and removal of pathogens by phagocytosis. However, pentraxin-3 cannot bind or recognize certain pathogens and microbial components such as LPS, lipoteichoic acid (LTA), enterotoxin A and B, exotoxin A, and N-Acetylmuramyl-L-Alanyl-D-Isoglutamine (54).

While the mechanisms mentioned earlier represent the most characterized mechanisms, additional mechanisms mediating the effects of pentraxin-3 have been described in the literature (58). Further investigations are warranted to examine and uncover new pathways activated by pentraxin-3 to understand its diverse physiological functions.

## Pentraxin-3 In Asthma

We and others extensively examined the specific role of pentraxin-3 in asthma. For instance, we previously reported the expression of pentraxin-3 in airway epithelium, infiltrating inflammatory cells, and airway smooth muscle (ASM) bundles of asthmatic bronchial biopsies (94). Among these cells, ASM cells have been shown to contribute significantly to airway hyperresponsiveness (AHR) and airway remodeling. AHR and airway remodeling cause the clinical manifestations of asthma (95) featured by smooth muscle outgrowth and hypercontraction, mucus hyper-secretion, and associated airway inflammation (24). The pathological mechanisms involved in these events include alteration of the underlying mesenchymal layer resulting in hyperplasia and hypertrophy of ASM and subepithelial fibrosis (96, 97).

### Pentraxin-3 Expression and Functions in ASM Cells

We were the first to examine the secretion of pentraxin-3 in ASM cells. For instance, cultured ASM cells secreted higher levels of pentraxin-3 than that of epithelial cells treatment, suggesting that ASM is a major source of pentraxin-3 in the airways (94). When ASM cells were treated with a variety of cytokines found in high levels in the airways of asthma such as TNFα, IL-1β, Th1 (IFN-γ), Th2 (IL-4, IL-9, and IL-13), and Th17 (IL-17) cytokines, only TNF and IL-1β were able to increase pentraxin-3 secretion significantly (73, 94, 98). Specifically, TNF-induced pentraxin-3 secretion in ASM cells in a dose-dependent manner, where a significant effect was seen as low as 0.1 ng/ml, and in a time-dependent manner with an effect sustained up to 48 h (98). Further studies in ASM cells examined whether pentraxin-3 induces the secretion of cytokines and chemokines. When ASM cells were treated with 50, 100, and 500 ng/ml of human recombinant pentraxin-3 for 24 h, a significant increase in the levels of eotaxin-1/CCL11 was seen. However, the levels of TGF-β1, IL-6, or CXCL8/IL-8 were not affected (94). Since TNF induces pentraxin-3 secretion and the later promotes the secretion of eotaxin-1/CCL11, it is legitimate to speculate that pentraxin-3, in an autocrine manner, mediates some of the effects of TNF in asthma.

Further studies examined the effect of pentraxin-3 on ASM proliferation and migration functions. FGF2 is a growth factor previously reported to be expressed at high levels in asthmatic airways and to contribute to airway remodeling *via* the regulation of ASM proliferation and migration (99, 100). When ASM cells were treated with FGF-2, pentraxin-3 significantly inhibited the ability of FGF2 to induce migration (101). This may be due to the ability of pentraxin-3 to bind FGF-2, thereby preventing its biological functions (74, 76). In contrast, pentraxin-3 did not interfere with the ability of FGF-2 to induced cell proliferation in ASM cells which is in disagreement with studies in vascular smooth muscle cells where pentraxin-3 inhibits FGF2-induced cell proliferation (76). These studies suggest that pentraxin-3 effects on cell proliferation are highly cell-specific and warrants further investigation.

### Pentraxin-3 Expression and Functions *in vivo*

Since pentraxin-3 expression is increased in many inflammatory diseases, it is now a well-accepted serological marker of disease severity. However, pentraxin-3 has been reported to protect against severe infections and be a central player in the immune and inflammatory responses (35). Hence, whether pentraxin-3 upregulation in inflammatory diseases exerts beneficial or detrimental effects remains, however, controversial.

We were the first to examine the role and function of pentraxin-3 in patients with asthma (94). We showed that pentraxin-3 expression was significantly higher in bronchial biopsies obtained from patients with allergic asthma than healthy donors. Pentraxin-3 levels were also considerably higher in bronchoalveolar lavage fluid (BALF) of patients with severe asthma (102). Upon allergen challenge, lung mesenchymal and immune cells in the lung (94) secrete pentraxin-3 (102), which in turn inhibits FGF-mediated ASM cell migration to the submucosa and reduces smooth muscle outgrowth and hypercontraction. Pentraxin-3 may also inhibit epithelial-mesenchymal transition (EMT) (103). For instance, pentraxin-3-overexpressing melanoma cells manifested a reversed transition from a mesenchymal to an epithelial-like appearance, inhibition of cell proliferation, reduced motility and invasive capacity, a down-regulation of various mesenchymal markers, and an upregulation of the epithelial marker E-cadherin (103), all which are features happening in allergic asthma. Also, pentraxin-3 binding to FGF-2 inhibits endothelial cell proliferation *in vitro* and angiogenesis *in vivo* (32, 74), suggesting that the beneficial effect of pentraxin-3 in airway remodeling may be also due to the reduction of lung neoangiogenesis, as recently demonstrated in a murine model of fibrosarcoma (104).

Moreover, pentraxin-3 physically interacts with TSG-6 through its N-terminal domain (74) during deposition of extracellular matrix (ECM) of cumulus oophorus, an essential step in ECM organization (105). Since TSG-6 deficiency protects against AHR after ozone *in vivo* exposure (106), hyaluronan (HA) pathological matrix deposition and AHR in an allergic murine airway inflammation model (107), it is tempting to speculate that the effect of pentraxin-3 on airway remodeling is through the inhibition of TSG-6 (Table 2).

**Table 2:** The effect of Pentraxin-3 in allergic asthma.

***In vitro***	**Pentraxin-3 gene deficiency**		**Plausible effects of pentraxin-3**
	***Ex vivo***	***In vivo***	
Inhibits FGF-2 induced ASM cell migration	Enhances CD4 T cell survival and Bcl2 expression	High level of IgE/IgG2a and Th17 cytokines	Reduces lung neo-angiogenesis
	High expression of Th17 polarizing cytokines (IL-6 and IL-23) in lung CD11c+CD11b+ DCs	Exaggerates airway eosinophilia and neutrophilia	Reduces epithelial-mesenchymal transition
	Low CD4 T cell IL-2 expression and increases STAT-3 activation	Exacerbates AHR and goblet cell hyperplasia	Inhibits TSG-6 mediated hyaluronan deposition in the lung

Further study in children has investigated whether pentraxin-3 level can discriminate between eosinophilic from non-eosinophilic asthma. Plasma concentrations were not different in the two asthma subgroups, whereas sputum pentraxin-3 levels were higher in non-eosinophilic asthma (108). In another study, sputum pentraxin-3 concentration was significantly higher in children with asthma than in control subjects and correlated with atopic status and disease severity among patients with asthma (109). Also, Licari et al. showed that pentraxin-3 is a valuable marker for the presence of a low-grade inflammatory status and a low-grade fibrinolytic activity in children with asthma but could not detect significant associations with asthma severity and asthma control (110).

Recently, we examined the impact of pentraxin-3 deletion on allergen-induced inflammation (Table 2). Using a murine model of allergic asthma, we found that pentraxin-3 deletion aggravated disease severity (102) where an increase in airway inflammation and airway hyperresponsiveness were observed. Interestingly, while the total inflammatory cells, BALF eosinophil and neutrophil counts and lung tissue eosinophilia were increased after allergen exposure irrespective of pentraxin-3 deletion, it was more prominent in pentraxin-3^-/-^ mice.

**Figure 1:**
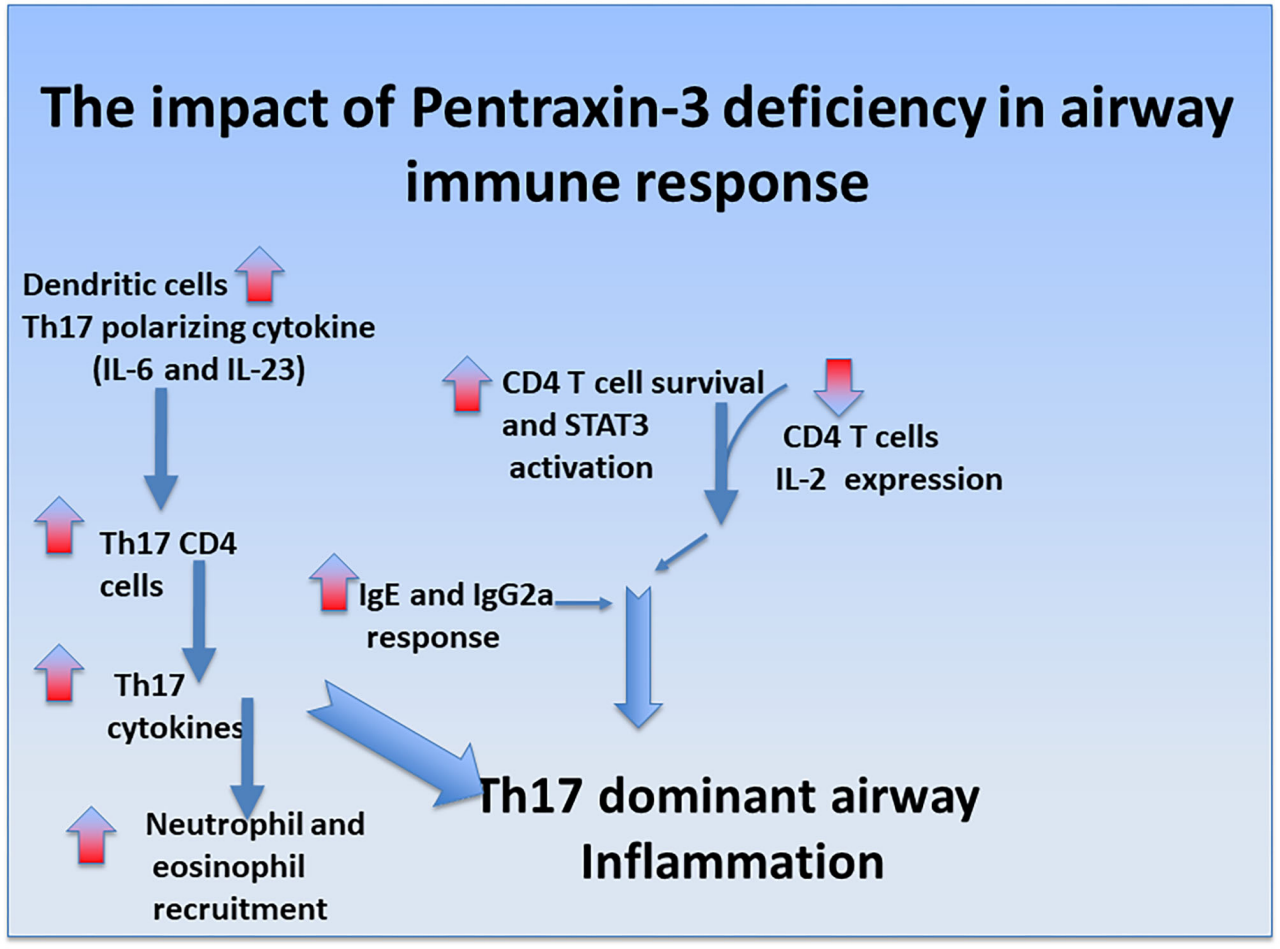
Regulatory role of pentraxin-3 in airway immune response. Ovalbumin exposure induces mixed neutrophilic and eosinophilic inflammation in pentraxin-3^-/-^ mice compared to pentraxin-3^+/+^ mice. It was also associated with CD4 T cells enhanced survival, heightened IgE/IgG2a response, and greater levels of Th17-polarizing cytokine IL-6, promoting Th17-dominant inflammation in pentraxin-3-depleted condition.

Pentraxin-3 has been reported to decrease neutrophil recruitment at sites of inflammation by binding to P-selectin (81). This suggests that pentraxin-3 may help prevent neutrophilic inflammation seen in patients with severe asthma. In line with this presumption, the number of neutrophils was higher in pentraxin-3^-/-^ mice than wild-type mice. Pentraxin-3^-/-^ mice also exhibited an enhanced neutrophil infiltration surrounding the airways and blood vessels, indicating a more severe inflammation (102). While pentraxin-3^-/-^ mice expressed lower levels of Th2 cytokines such as IL-4 and IL-5 than pentraxin-3^+/+^ mice, pentraxin-3^-/-^ mice expressed higher levels of IL-17A cytokines, indicating a dominant Th17 phenotype in the pentraxin-3^-/-^ mice (102). This phenotype dominance was further confirmed by the high levels of the Th17 pro-inflammatory cytokines such as IL-6 and IL-23 secreted by lung DCs obtained from pentraxin-3^-/-^ mice. Th17 response is usually associated with increased neutrophilic inflammation (as seen in pentraxin-3^-/-^ mice) and, thus, more severe asthma. These findings suggest that pentraxin-3 deletion promotes Th17 immune response (Figure 1), which has previously been associated with steroid-resistance seen in patients with severe asthma (111). Our observations are in concert with reported reduced levels of IL-17A upon treatment with exogenous pentraxin-3 in murine models of chronic *P. aeruginosa* lung infection (112) and cystic fibrosis (113) and suggest an inverse correlation between pentraxin-3 and IL-17A dependent pathway. Other studies showed that pentraxin-3 suppressed IL-17A-mediated inflammation in aspergillus-induced chronic granulomatous disease (114) and ischemia-reperfusion injury model (115) by restricting γδT cells expansion. Altogether, these studies demonstrated that pentraxin-3 plays a protective role in allergic asthma through, at least in part, interfering with Th17 phenotypes and associated pathways.

## Conclusion

**Figure 2:**
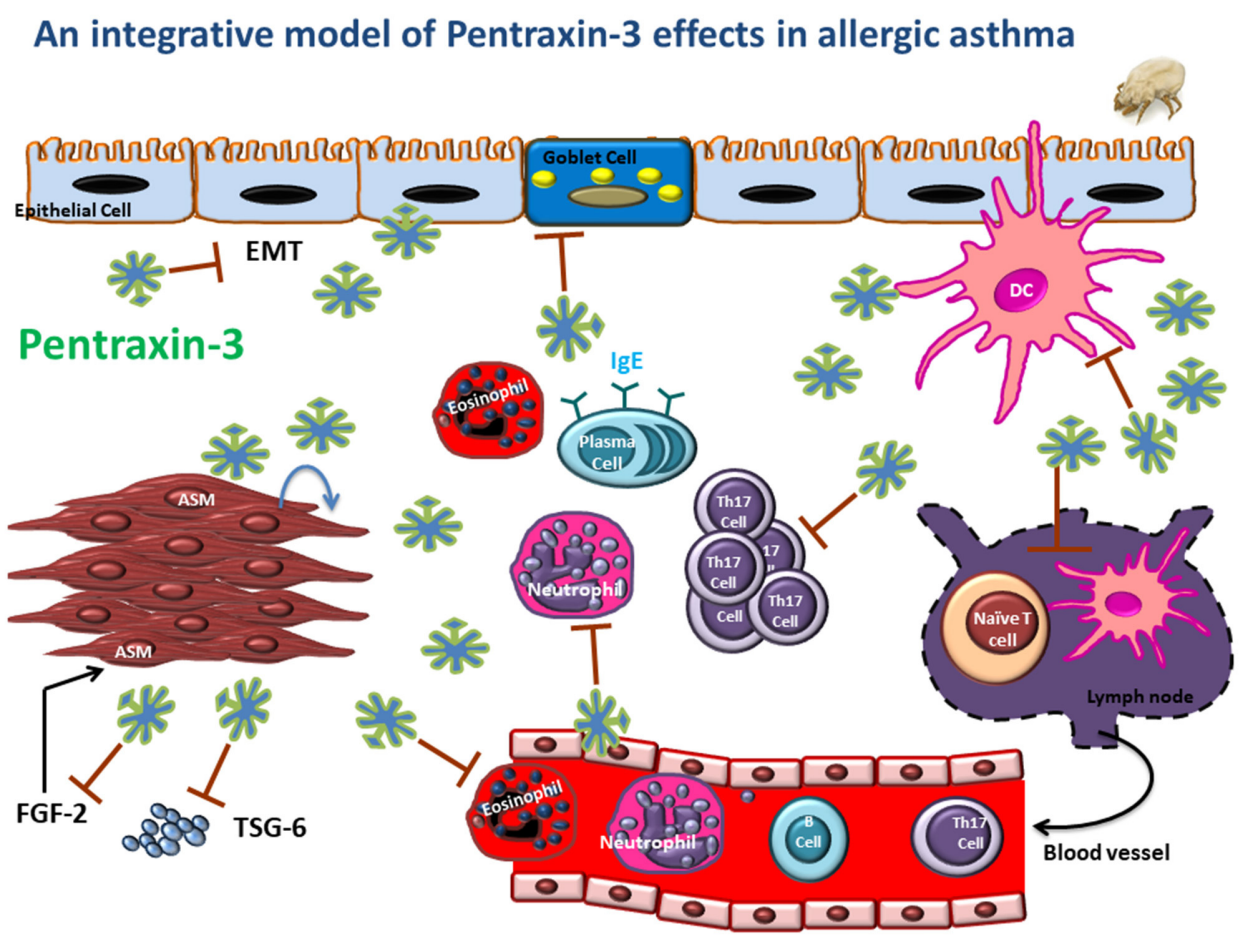
An integrative model is depicting the potential regulatory role of pentraxin-3 in asthmatic airways. Pentraxin-3 produced locally can regulate airway inflammatory response by targeting many pathways. (i) reducing DC Th17 polarizing cytokine (IL-6 and IL-23), leading to attenuated Th17 cytokine response; (ii) inhibiting neutrophil and eosinophil recruitment by binding to P-selectin on endothelial cells; (iii) downregulating goblet cell hyperplasia and IgE response by to be determined mechanism. Also, pentraxin-3 may affect epithelial-mesenchymal transition (EMT), ASM cell migration and matrix deposition through interaction with FGF2 and TSG-6, thus reducing exaggerated AHR and tissue remodeling.

Pentraxin-3 is a soluble pattern recognition receptor that acts as a significant regulator of innate immune responses. In allergic asthma, it is produced by both immune and structural cells, and its upregulation is induced by pro-asthmatic cytokines such as TNF and IL-1β. Traditional views described pentraxin-3 as a marker of the severity of many inflammatory diseases. However, emerging evidence now demonstrated the protective role of pentraxin-3 in chronic airway inflammatory diseases such as allergic asthma. Indeed, pentraxin-3 deletion promotes more severe forms of asthma characterized mainly by a steroid-resistant Th17 dominant phenotype (Figure 2). Further studies, however, are still needed to examine whether:

i) pentraxin-3 exerts its protective effects in allergic asthma, at least in part, by interfering IL17 signaling pathways.
ii) the ability of GCs to increase the expression of pentraxin-3 in ASM cells is defective in patients with severe asthma.
iii) different pentraxin-3 isoforms are expressed in patients with severe asthma to explain why the high levels of pentraxin-3 seen in these patients are not protective in deterring the inflammatory response.

## Author Contributions

All the authors have contributed to writing, editing, and researching the literature for this manuscript.

## Conflict of Interest

The authors declare that the research was conducted in the absence of any commercial or financial relationships that could be construed as a potential conflict of interest.
